# Epidemiological Characteristics of Classical Scrapie Outbreaks in 30 Sheep Flocks in the United Kingdom

**DOI:** 10.1371/journal.pone.0003994

**Published:** 2008-12-22

**Authors:** K. Marie McIntyre, Simon Gubbins, Wilfred Goldmann, Nora Hunter, Matthew Baylis

**Affiliations:** 1 Pirbright Laboratory, Institute for Animal Health, Pirbright, Surrey, United Kingdom; 2 Neuropathogenesis Division, The Roslin Institute and R(D)SVS, University of Edinburgh, Edinburgh, United States of America; 3 Leahurst Campus, University of Liverpool, Neston, Wirral, Cheshire, United Kingdom; Fred Hutchinson Cancer Research Center, United States of America

## Abstract

**Background:**

Most previous analyses of scrapie outbreaks have focused on flocks run by research institutes, which may not reflect the field situation. Within this study, we attempt to rectify this deficit by describing the epidemiological characteristics of 30 sheep flocks naturally-infected with classical scrapie, and by exploring possible underlying causes of variation in the characteristics between flocks, including flock-level prion protein (*PrP*) genotype profile. In total, the study involved *PrP* genotype data for nearly 8600 animals and over 400 scrapie cases.

**Methodology/Principal Findings:**

We found that most scrapie cases were restricted to just two *PrP* genotypes (ARQ/VRQ and VRQ/VRQ), though two flocks had markedly different affected genotypes, despite having similar underlying genotype profiles to other flocks of the same breed; we identified differences amongst flocks in the age of cases of certain *PrP* genotypes; we found that the age-at-onset of clinical signs depended on peak incidence and flock type; we found evidence that purchasing infected animals is an important means of introducing scrapie to a flock; we found some evidence that flock-level *PrP* genotype profile and flock size account for variation in outbreak characteristics; identified seasonality in cases associated with lambing time in certain flocks; and we identified one case that was homozygous for phenylalanine at codon 141, a polymorphism associated with a very high risk of atypical scrapie, and 28 cases that were heterozygous at this codon.

**Conclusions/Significance:**

This paper presents the largest study to date on commercially-run sheep flocks naturally-infected with classical scrapie, involving 30 study flocks, more than 400 scrapie cases and over 8500 *PrP* genotypes. We show that some of the observed variation in epidemiological characteristics between farms is related to differences in their *PrP* genotype profile; although much remains unexplained and may instead be attributed to the stochastic nature of scrapie dynamics.

## Introduction

Scrapie is a fatal neurodegenerative disorder of sheep and goats within the transmissible spongiform encephalopathy (TSE) group of diseases, which includes variant Creutzfeldt-Jakob disease (vCJD) and kuru in humans, bovine spongiform encephalopathy (BSE) in cattle and chronic wasting disease (CWD) in mule deer and elk. Scrapie has been present in British sheep for centuries [1], [2], but increased interest has been stimulated in recent years by several factors. First, the clinical signs of scrapie are similar to those of experimental BSE in sheep [3] raising the possibility that scrapie has obscured BSE cases in the UK sheep population. Second, new strains of scrapie have been identified recently, such as Nor98 [4]. Finally, a host genetic component, the ovine prion protein (*PrP*) gene, strongly affects progression to clinical disease and the incubation period (IP), such that some sheep *PrP* alleles confer resistance or longer IPs, while others confer susceptibility or shorter IPs [5]–[8]. This discovery has allowed the possibility of genetic-based selective breeding programmes to control scrapie, and such programmes are now implemented across the European Union (EU).

With few exceptions [9]–[11], previous analyses of scrapie outbreaks have focused on those in flocks run by research institutes [12]–[17], which have the advantage of facilitating detailed study, but do not necessarily reflect the field situation. Within this study, we attempt to rectify this deficit, by describing the epidemiological characteristics of 30 sheep flocks naturally-infected with classical scrapie, which formed part of a large farm-based case-control study of scrapie in sheep flocks undertaken by the Institute for Animal Health (IAH) since 1998. Furthermore, we explore possible underlying causes of variation in the epidemiological characteristics of outbreaks, including flock-level *PrP* profile.

The effects of the ovine *PrP* gene are most apparent at the ‘individual-level’: a sheep does or does not get scrapie. However, there is significant variation in the frequencies of the different *PrP* alleles amongst flocks (especially of different breed; [5]), raising the possibility that the pattern of genotypes present in a flock may have flock-level effects on scrapie epidemiology and, in particular, measures of outbreak size or scale, such as incidence and duration. Between-flock variation in *PrP* genotype frequency has already been identified as a flock-level risk factor for the occurrence of scrapie [10]; however, the effects of *PrP* genotype and other flock characteristics on epidemiological parameters such as outbreak duration or the incidence of clinical disease have not yet been reported. Other causes of variation in disease occurrence include scrapie strain, flock management practices and demography or, simply, the stochastic nature of infectious disease dynamics, which can differ even when the underlying parameters are similar [18]–[20]. We investigate whether flock-level, *PrP*-based risk factors can be identified which account for the wide variation in outbreak sizes seen across the flocks, or whether the variation is better attributed to stochasticity in disease dynamics.

## Materials and Methods

### Flocks and cases

Flocks were eligible to join the IAH study if they had had at least one case of classical scrapie confirmed in the previous two years. Upon recruitment, all subsequent cases were reported by the farmers to the relevant authorities and suspect animals were confiscated. Tissue samples were sent to the Veterinary Laboratories Agency (VLA) for routine analysis for evidence of classical scrapie [21]. Importantly, all the cases would have been showing clinical signs of disease and were confirmed by laboratory diagnosis using methods which would not have misidentified these animals as having atypical scrapie. Upon confiscation, demographic data for cases including the animal's breed, sex, date of birth, date of death, *PrP* genotype and whether the case occurred in a homebred or purchased animal were recorded in the Scrapie Notification Database (SND) held at the VLA. Analysis of cases includes those of both homebred and purchased origin. It is important to note that in several flocks, cases had been reported for a number of years before joining the IAH study; here we use all the data on scrapie cases in our flocks held in the SND (both before and after joining the IAH study) and therefore report on the entire, officially-confirmed outbreaks within the flocks. Of the 415 scrapie cases that were reported in these flocks, 327 (79%) were genotyped.

### Flock-level demographic data

After agreeing to join the IAH study and providing evidence of confirmation of a scrapie case within the appropriate time period, flock-level data were collected by IAH staff. Field visits were undertaken and the entire breeding flock was blood sampled for *PrP* genotyping. Questionnaires, which were completed by the farmers, were used to collect farm and flock characteristics and additional data on the history of the outbreaks, including the origin of the first case of scrapie in their flocks and the clinical signs of disease. Questionnaires were returned by farmers to IAH before the provision of *PrP* genotypes, and in most cases this was six to twelve months after blood sampling.

### PrP genotype analysis

Blood samples (5 ml) were collected from the entire breeding stock (all sheep over approximately one year of age) of the 30 affected flocks (n = 8595) and genotyped according to published methods [22]. In addition to the standard polymorphisms at codons 136, 154 and 171, the frequencies of amino acid changes at codons 112 (methionine to threonine), 141 (leucine to phenylalanine), 168 (proline to leucine) and 241 (proline to serine) were also examined for 11 flocks in which samples were available.

### Flock-level susceptibility

Genotypes of the individual sheep within a flock were combined to provide a number of flock-level indicators of susceptibility (table 1). The simplest, *s_sus_*, is the proportion of the flock that is of the most highly susceptible genotypes (National Scrapie Plan (NSP) type 5; see table 1). The second indicator, *s_res_*, is the proportion of the flock that is not of the most highly resistant genotype (i.e. not ARR/ARR). The third indicator, *s_risk_*, is based on the relative frequency of genotypes in the flock weighted according to the risk of scrapie in that genotype, so that,
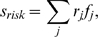
where *f_j_* is the proportion of the flock of genotype *j* and *r_j_* is the estimate for risk of scrapie in that genotype relative to VRQ/VRQ [23]. The risk estimates, *r_j_*, were derived from the ‘high susceptibility’ estimates presented in [24], based on data from [6]. These are essentially estimates of risk averaged at a national level and, therefore, reflect the high frequency of VRQ-type scrapie (scrapie strains that target sheep encoding the VRQ allele in UK affected flocks). In a smaller number of outbreaks, ARQ-type scrapie causes significant levels of disease (and hence, high apparent susceptibility) in sheep that encode the ARQ allele, but not the VRQ allele. In the current study, one flock (flock 21) showed evidence of a strain of scrapie able to target such ARQ, non-VRQ sheep to a significant extent.

**Table 1:** Definitions of epidemiological parameters used in the study.

term	symbol	definition
flock size	*N*	number of ewes and rams over one year old at time of blood sampling
total number of cases	*C*	number of confirmed clinical cases during the outbreak
outbreak duration	*D*	time between the first and last confirmed case in the outbreak
mean incidence	*I*	*C*/(*N*×number of years with cases)
peak incidence	*I*_max_	(maximum number of cases during a twelve month period)/*N*
outbreak size	*S*	*C/N*
age-at-onset	-	difference between date of birth and date when animal reported as clinical suspect
flock-level susceptibility	*s_sus_*	proportion of sheep that are NSP type 5 (i.e. AHQ/VRQ, ARH/VRQ, ARQ/VRQ and VRQ/VRQ)
	*s_res_*	proportion of sheep that are not ARR/ARR
	*s_risk_*	sum of the frequency of each genotype weighted by its susceptibility (see methods for details)

### Statistical methods

A number of epidemiological parameters were used to characterise the outbreaks: number of cases; outbreak size (number of cases divided by flock size); mean incidence; peak incidence over a 12 month period; outbreak duration (time between the first and last confirmed cases in the flock); and age-at-onset of cases. These parameters are defined in table 1. The effects of changes in flock size over the course of scrapie epidemics upon the mean and peak incidence parameters were examined, but as this did not cause major changes in the results or conclusions of analyses, a single point estimate for flock size was utilised (that at the time of blood sampling).

#### Outbreak duration

A Cox proportional hazard model [25] with duration as the survival measure was used to examine the effects of the measures of flock-level susceptibility, flock size and flock type upon outbreak duration. The different flock types (commercial versus purebred) reflect different husbandry practices: the primary business of commercial flocks is production of slaughter lambs, while for purebred flocks it is production of animals for further breeding. Outbreak duration was not defined for flocks with singleton cases. However, we assume that such flocks have outbreaks of very short duration and we do not want to lose this information from analysis. A useful approach to avoid this is winsorization [26], in which the outliers in an ordered array are replaced by the next closest value so that they still contribute to the sample size but are less extreme and have less leverage. Outbreak durations for singleton flocks were set to 0.1; the lowest value for flocks with more than a single case.

#### Disease incidence

Linear models were used to assess the effects of the measures of flock-level susceptibility, flock size and flock type upon mean incidence, peak incidence and outbreak size. Mean incidence and peak incidence were log-transformed to normalise the data; a logit transformation was used for outbreak size. For each measure of disease incidence and flock susceptibility, model construction proceeded by stepwise deletion of non-significant terms from an initial model including flock-level susceptibility, flock size and flock type. Flock 21 was excluded from the analysis of outbreak size because its value for this characteristic was 3.4 standard deviations from the mean. Flocks 1 and 7 were missing information on flock type, but because this factor was never significant, these flocks were included in subsequent analyses.

#### Age-at-onset

Differences in the age-at-onset of clinical signs amongst flocks and *PrP* genotypes were assessed using Wilcoxon tests [25]. Where significant differences in age at onset for a *PrP* genotype were identified amongst flocks, Cox proportional hazard models [25] with age-at-onset of clinical signs as the survival measure were used to explore whether mean and peak incidence, outbreak size, outbreak duration and flock type explained these differences.

#### Seasonality

Chi-squared tests were used to see if the observed number of cases per quarter in individual flocks were uniformly distributed (i.e. the expected number of cases in each quarter was equal to the total number of cases divided by four). A Bonferroni correction for multiple testing was used in this analysis.

## Results

The 30 flocks are spread across the UK, are composed of different sheep breeds and vary in size from about fifty to eight hundred animals. There was considerable variability in their epidemiological characteristics (table 2). In the subsequent sections, we explore how the epidemics were similar or dissimilar, and how large and small outbreaks differed in terms of the epidemiological parameters listed in table 1.

**Table 2:** Epidemiological parameters for scrapie outbreaks in 30 sheep flocks.

flock	main breed*	flock size (*N*)	flock type†	year of first case	no. cases (*C*)	origin of cases‡	outbreak duration (*D*, years)	mean incidence (*I*)	peak incidence (*I*_max_)	outbreak size (*S*)	flock-level susceptibility		
						%P,%H					*s_sus_*	*s_res_*	*s_risk_*
1	BWM	267	-	1996	15	0,33	2.5	1.9	3.4	5.6	0.11	0.70	0.04
2	BHC	255	C	1998	3	0,100	0.3	1.2	1.2	1.2	0.06	0.80	0.03
3	BHC	542	PC	1998	15	0,100	2.2	0.9	2.0	2.8	0.12	0.85	0.05
4	Cha	73	P	1997	1	0,0	0.1	1.4	1.4	1.4	0.08	0.82	0.06
5	Cha	56	C	2000	1	0,100	0.1	1.8	1.8	1.8	0.07	0.77	0.03
6	Cha	144	P	1997	1	0,0	0.1	0.7	0.7	0.7	0.07	0.88	0.05
7	Cha	427	-	1995	2	0,0	1.4	0.2	0.5	0.5	0.07	0.80	0.04
8	C	392	PC	2000	1	0,100	0.1	0.3	0.3	0.3	0.06	0.80	0.03
9	FD	698	C	1998	18	0,100	5.4	0.4	1.4	2.6	0.09	0.78	0.05
10	F	494	P	1997	12	0,83	4.6	0.5	0.8	2.4	0.17	0.99	0.06
11	F	320	PC	1999	18	0,100	3.3	1.4	3.4	5.6	0.30	0.91	0.14
12	G	59	P	1995	1	0,0	0.1	1.7	1.7	1.7	0.08	0.98	0.08
13	L	90	P	1998	2	50,0	0.3	2.2	2.2	2.2	0.02	0.66	0.01
14	NCC	194	PC	1993	6	0,33	6.8	0.4	1.0	3.1	0.06	0.79	0.04
15	NEM	471	C	2002	2	50,50	1.9	0.2	0.2	0.4	0.10	0.87	0.05
16	PD	493	PC	1998	9	0,100	1.1	0.9	1.6	1.8	0.06	0.72	0.04
17	PD	190	P	1998	10	0,100	1.4	2.6	3.7	5.3	0.20	0.89	0.12
18	PD	306	P	1993	19	0,32	7.2	0.8	2.3	6.2	0.04	0.53	0.02
19	R	114	PC	1999	2	0,50	0.1	1.8	1.8	1.8	0.06	0.91	0.05
20	Sh	228	C	2001	5	0,100	1.3	1.1	1.8	2.2	0.08	0.90	0.06
21	Sh	132	C	2000	29	90,10	4.1	4.4	12.9	22.0	0.04	0.91	0.04
22	Sh	496	C	1993	30	0,37	9.0	0.6	1.8	6.0	0.07	0.95	0.06
23	Sh×C	61	C	1999	8	0,100	2.6	4.4	9.8	13.1	0.15	0.98	0.10
24	Swa	426	C	1994	44	0,93	6.7	1.5	6.3	10.3	0.10	0.84	0.03
25	T	71	P	2002	1	100,0	0.1	1.4	1.4	1.4	0.08	0.89	0.04
26	T	202	P	1997	1	0,0	0.1	0.5	0.5	0.5	0.04	0.96	0.03
27	T	212	PC	1994	2	0,50	7.4	0.1	0.5	0.9	0.01	0.91	0.02
28	T	131	P	1994	5	0,40	5.2	0.6	2.3	3.8	0.00	0.95	0.01
29	T	234	P	1998	21	0,100	1.9	4.5	5.6	9.0	0.09	0.96	0.07
30	WM	817	PC	1997	131	0,87	6.4	2.3	6.1	16.0	0.14	0.84	0.05

* breeds are: Black Welsh Mountain (BWM); Brecknock Hill Cheviot (BHC); Charollais (Cha); Cheviot (C); Finn Dorset (FD); Friesland (F); Gotland (G); Lleyn (L); North Country Cheviot (NCC); North of England Mule (NEM); Poll Dorset (PD); Roussin (R); Shetland (Sh); Swaledale (Swa); Texel (T); Welsh Mountain (WM).

† purebred (P), commercial (C) or mixed purebred and commercial (PC).

‡ purchased (P), homebred (H); the origin of the remaining of cases is unknown.

### Origin of outbreaks and cases

**Figure 1:**
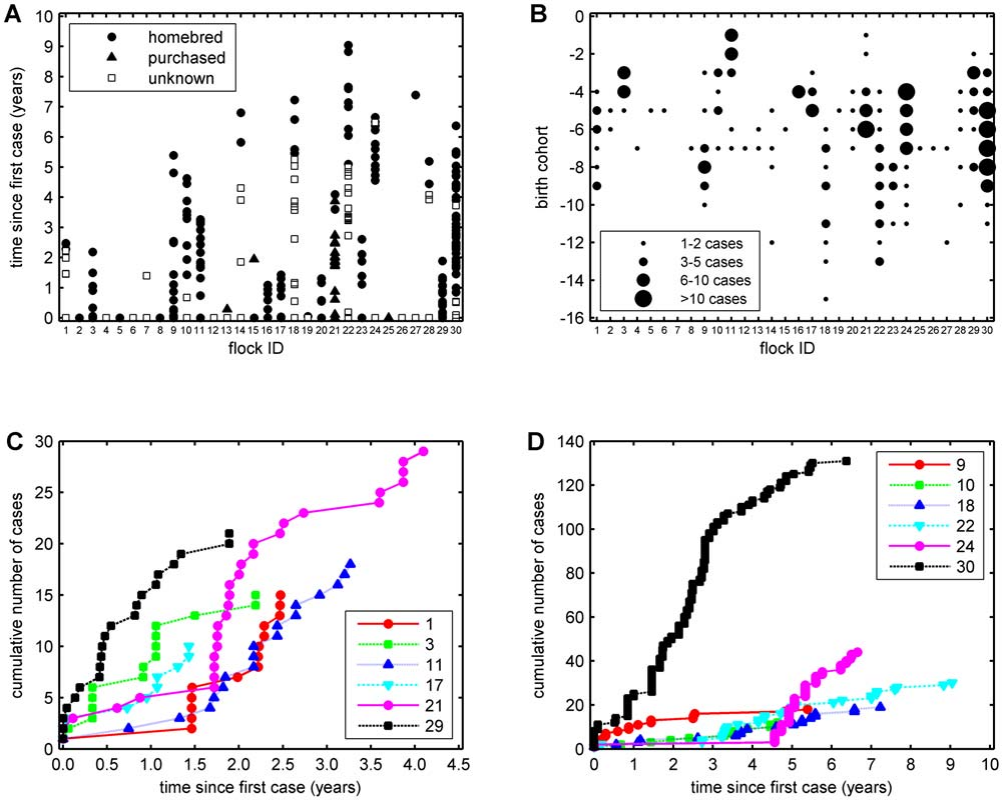
Time-course of scrapie outbreaks in 30 sheep flocks in the UK. (a) Occurrence and origin of cases within each outbreak. Time zero corresponds to the first case of scrapie in each flock. The origin of cases is that recorded in the Scrapie Notifications Database (SND). (b) Frequency of cases by birth cohort. Time zero corresponds to the first birth cohort for which the farmer had received genotype results from the IAH. The date that the farmer of flock 7 was informed of his genotypes was unknown and is not displayed. (c,d) Epidemic curves showing the cumulative number of cases in (c) outbreaks <50 months long; and (d) outbreaks >50 months long. Legends indicate flock identification number.

In the questionnaire, 68% (n = 19) of farmers reported that the first case of scrapie in their flock had been in a homebred animal, and 32% (n = 9) reported the first case in a purchased animal. Two farmers did not provide this information. Considering all, as opposed to just first cases, however, 306 (91%) were recorded in SND as homebred animals and 29 (9%) were purchased animals; only two flocks had cases in both homebred and purchased animals (figure 1a). The origins of 80 cases (19%) were not recorded, possibly because specific details for animals were not known by the farmer. The proportion of first cases that were purchased, as reported by farmers, is significantly higher than the proportion of all cases that were purchased, as recorded on SND (χ^2^ = 15.2, df = 1, *P*<0.001).

### Outbreak duration

The duration of epidemics in the 23 flocks with more than one case varied substantially (figure 1a), with a maximum, mean and median duration of 9, 3.6 and 2.6 years, respectively.

There were often gaps of several months between cases being reported within a flock (figure 1a), and in certain examples these gaps exceeded two years (figure 1a). Moreover, no cases of scrapie occurred in animals born after farmers received their *PrP* genotype results (figure 1b).

There was no association between outbreak duration and any measure of flock-level susceptibility. Flock type was significantly associated with outbreak duration (winsorized data for singleton flocks; P<0.001), with purebred flocks more likely to experience longer epidemics than either commercial (hazard ratio, HR = 0.54) or mixed purebred and commercial flocks (HR = 0.59). The effect of flock size upon duration was significant at the 10% level (winsorized data for singleton flocks, *P* = 0.08), such that larger flocks might experience longer epidemics. Changing the outbreak duration for winsorized flocks from 0.1 to 0.2 did not affect the conclusions of the analysis.

### Epidemic curves

Epidemic curves were drawn showing the cumulative number of cases for flocks in which there were at least ten cases (n = 12). The outbreaks were split into two different groups for ease of plotting; those lasting under four years (figure 1c) and those lasting over four years (figure 1d). Two different types of epidemic were identified: those with cases occurring at relatively regular intervals (flocks 3, 9, 17, 29 and 30); and those which had one or two cases over the course of a few years, after which the epidemic accelerated and cases were reported at more regular intervals (flocks 1, 10, 11, 18, 21, 22 and 24).

### Disease incidence

**Figure 2:**
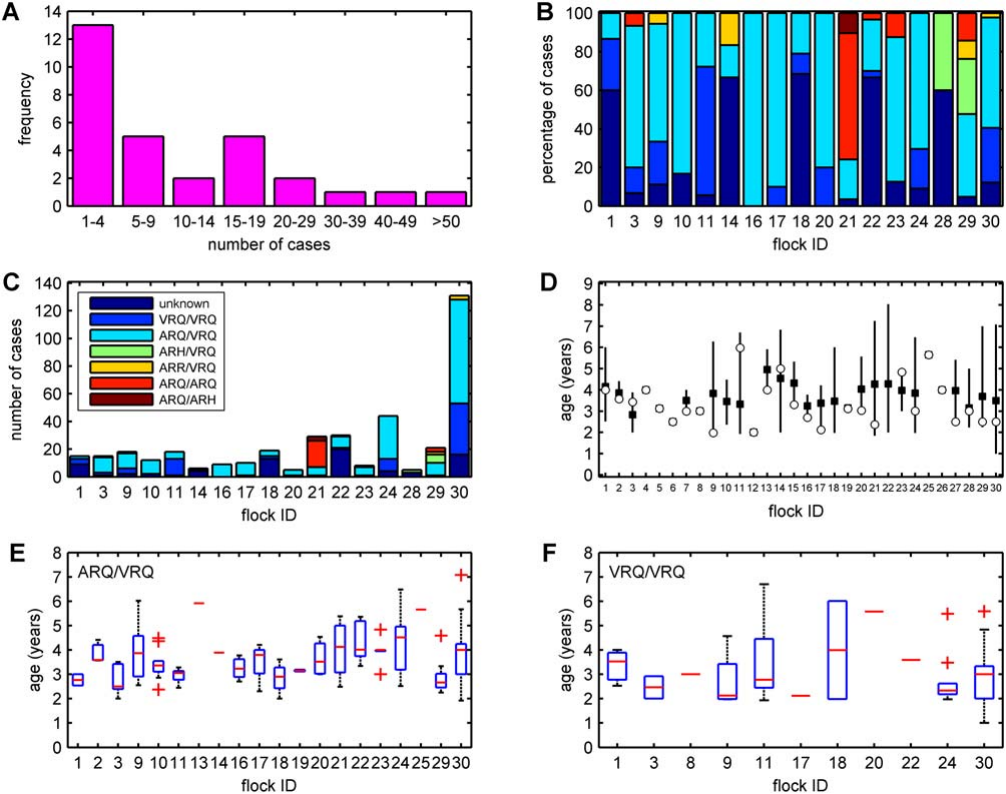
Epidemiological characteristics of scrapie outbreaks in 30 sheep flocks in the UK. (a) Distribution of the number of cases. (b,c) *PrP* genotypes of cases in flocks with at least five cases of scrapie: (b) proportion (%) of cases by genotype; and (c) frequency of cases by genotype. Genotypes are indicated by the legend in figure (c). (d) Age-at-onset of scrapie cases: mean (black squares) and range (error bars) for age-at-onset, and age of first case (white circle). (e,f) Box and whisker plots for the age at onset in (e) ARQ/VRQ and (f) VRQ/VRQ animals in individual flocks. The boxes show the lower quartile, median and upper quartile; the whiskers represent 1.5 times the interquartile range; and the crosses indicate any outlying values.

In total, 415 cases of scrapie were confirmed in the 30 flocks, with numbers of cases per flock varying from 1 to 131 (figure 2a). Seven flocks had only a single case of scrapie. The mean incidence varied from 0.1 to 4.5 cases per 100 animals per year (table 2; mean = 1.4, median = 1.1), while the peak incidence varied between flocks from 0.2 to 12.9 cases per 100 animals per year (table 2; mean = 2.7, median = 1.8). Finally, outbreak size varied between flocks from 0.3 to 22.0 cases per 100 animals (table 2; mean = 4.4, median = 2.3).

The mean incidence of disease was significantly higher in flocks with a higher level of susceptibility (as measured by *s_risk_*) (*b* = 12.3, *F*_1,28_ = 4.8, *P* = 0.04). With *s_risk_* included in the model, the effect of flock size upon mean incidence was significant at the 10% level (*b* = −0.002, *F*_1,27_ = 3.4, *P* = 0.08), while flock type did not explain further variation in mean incidence (*P* = 0.34). Similarly, the peak incidence was greater in flocks with a higher susceptibility (as measured by *s_risk_*) (*b* = 10.6, *F*_1,28_ = 3.1, *P* = 0.09). However, flock size or flock type did not explain further variation in peak incidence (*P* = 0.66 and *P* = 0.54, respectively). In the models including the flock susceptibility measures, *s_sus_* and *s_res_*, none of the terms (flock susceptibility, flock size and flock type) were significantly associated with mean or peak incidence of disease.

Flocks of greater flock susceptibility tended to have larger outbreaks; this was significant at the 10% level for *s_risk_* (*b* = 0.13, *F*_1,27_ = 3.2, *P* = 0.08), and was significant at the 5% level for *s_sus_* (*b* = 0.08, *F*_1,27_ = 4.9, *P* = 0.04), but was not significant for *s_res_* (*P* = 0.88). Neither flock size nor flock type explained further variation in any of the models.

### PrP genotype of cases

Confirmed cases occurred in six (out of 15) *PrP* genotypes, predominantly those encoding VRQ. The most common genotypes of cases were ARQ/VRQ (n = 208) and VRQ/VRQ (n = 74), and together these comprised 88% of the cases for which genotypes are known (figure 2b). The proportion of cases in each genotype varied by flock (table 3, figure 2c). In most flocks, cases were confined to the two most common genotypes: ARQ/VRQ and VRQ/VRQ. Some flocks, however, had markedly different affected genotypes (e.g. flock 21 and, to a lesser extent, flock 29), with a substantially larger proportion of cases in animals of the ARQ/ARQ and ARQ/ARH genotypes. Moreover, these flocks had similar underlying *PrP* genotype profiles to other flocks of the same breed (table 3).

**Table 3:** Number of cases in each *PrP* genotype, and the percentage of each *PrP* genotype in the flock at the time of blood sampling (in brackets).

flock	main breed*	ARR/ARR	ARR/AHQ	ARR/ARH	ARR/ARQ	AHQ/AHQ	AHQ/ARH	AHQ/ARQ	ARH/ARH	ARQ/ARH	ARQ/ARQ	ARR/VRQ	AHQ/VRQ	ARH/VRQ	ARQ/VRQ	VRQ/VRQ	UNK†
1	BWM	− (30.3)	− (8.2)	− (0)	− (9.4)	− (0.4)	− (0)	− (3)	− (0)	− (0)	− (0.7)	− (37.1)	− (5.6)	− (0)	2 (3.4)	4 (1.9)	9
2	BHC	− (20.8)	− (12.6)	− (0)	− (28.3)	− (1.1)	− (0.7)	− (10.4)	− (0)	− (1.9)	− (11.5)	− (7.1)	− (2.6)	− (0)	3 (3)	− (0)	0
3	BHC	− (15.5)	− (13.5)	− (0)	− (22)	− (2.6)	− (0)	− (10.5)	− (0)	− (0)	1 (16.4)	− (7.9)	− (5)	− (0)	11 (5.9)	2 (0.7)	1
4	Cha	− (17.8)	− (0)	− (0)	− (43.8)	− (0)	− (0)	− (0)	− (0)	− (0)	− (12.3)	− (17.8)	− (0)	− (0)	− (5.5)	− (2.7)	1
5	Cha	− (23.2)	− (3.6)	− (12.5)	− (26.8)	− (1.8)	− (1.8)	− (1.8)	− (0)	− (0)	− (1.8)	− (19.6)	− (1.8)	− (1.8)	− (3.6)	− (0)	1
6	Cha	− (12.5)	− (0)	− (0)	− (47.9)	− (0)	− (0)	− (0)	− (0)	− (0)	− (28.5)	− (4.2)	− (0)	− (0)	− (6.9)	− (0)	1
7	Cha	− (20.4)	− (4.7)	− (1.4)	− (33.7)	− (0.2)	− (0.2)	− (3.3)	− (2.5)	− (0.2)	− (18)	− (0)	− (1.2)	− (0.5)	− (16.2)	− (0)	2
8	C	− (19.9)	− (18.6)	− (3.3)	− (19.6)	− (2.6)	− (3.1)	− (8.4)	− (0.3)	− (4.3)	− (8.9)	− (5.4)	− (2.6)	− (0.3)	− (2)	1 (0.8)	0
9	FD	− (22.2)	− (3.7)	− (0)	− (34.5)	− (0.1)	− (0)	− (4.3)	− (0)	− (0.1)	− (12.5)	1 (13.2)	− (1.9)	− (0)	11 (6.4)	4 (1)	2
10	F	− (0.4)	− (4.5)	− (0.2)	− (3.4)	− (21)	− (1.6)	− (33.6)	− (0)	− (1.6)	− (15.2)	− (0.7)	− (9.4)	− (0.2)	10 (7.8)	− (0.4)	2
11	F	− (11.3)	− (8)	− (2.3)	− (11.8)	− (3.3)	− (1)	− (14.5)	− (2.5)	− (3.8)	− (4.8)	− (13.3)	− (5.8)	− (0.3)	5 (12.8)	12 (5)	1
12	G	− (1.7)	− (0)	− (0)	− (22)	− (0)	− (0)	− (0)	− (0)	− (0)	− (61)	− (6.8)	− (0)	− (0)	− (8.5)	− (0)	1
13	L	− (34.4)	− (24.4)	− (4.4)	− (14.4)	− (5.6)	− (1.1)	− (3.3)	− (0)	− (4.4)	− (3.3)	− (2.2)	− (0)	− (0)	1 (2.2)	− (0)	1
14	NCC	− (21.1)	− (4.6)	− (2.6)	− (30.4)	− (0)	− (0.5)	− (8.8)	− (0)	− (1.5)	− (14.4)	1 (10.3)	− (0.5)	− (0)	1 (4.6)	− (0.5)	4
15	M	− (12.7)	− (8.7)	− (0.2)	− (33.3)	− (2.5)	− (0)	− (10)	− (0)	− (0)	2 (14.9)	− (8.1)	− (2.3)	− (0.6)	− (6.4)	− (0.2)	0
16	PD	− (27.6)	− (0)	− (0)	− (37.1)	− (0)	− (0)	− (0.2)	− (0)	− (0.2)	− (23)	− (5.8)	− (0)	− (0)	9 (6)	− (0)	0
17	PD	− (10.5)	− (0)	− (0)	− (30.5)	− (0)	− (0)	− (0)	− (0)	− (0)	− (21.1)	− (17.9)	− (0)	− (0)	9 (16.8)	1 (3.2)	0
18	PD	− (47.1)	− (0)	− (0)	− (35.9)	− (0)	− (0)	− (0)	− (0)	− (0)	− (5.2)	− (7.8)	− (0)	− (0)	4 (3.6)	2 (0.3)	13
19	R	− (8.8)	− (0)	− (1.8)	− (43.9)	− (0)	− (0)	− (0)	− (0)	− (0)	− (34.2)	− (5.3)	− (0)	− (0.9)	2 (5.3)	− (0)	0
20	Sh	− (10.1)	− (1.3)	− (0.4)	− (30.7)	− (0)	− (0)	− (3.5)	− (0)	− (0)	− (37.7)	− (8.3)	− (0)	− (0)	4 (7.9)	1 (0)	0
21	Sh	− (9.1)	− (3)	− (3)	− (36.4)	− (0)	− (1.5)	− (6.8)	− (0)	3 (3)	19 (25)	− (8.3)	− (0)	− (0)	6 (3.8)	− (0)	1
22	Sh	− (4.8)	− (3.6)	− (0)	− (38.5)	− (1.8)	− (0.2)	− (4)	− (0)	− (0)	1 (35.5)	− (4.6)	− (0.2)	− (0)	8 (6.7)	1 (0)	20
23	Sh×C	− (1.6)	− (0)	− (0)	− (16.4)	− (0)	− (0)	− (14.8)	− (0)	− (0)	1 (52.5)	− (0)	− (0)	− (0)	6 (14.8)	− (0)	1
24	Swa	− (16.2)	− (11.1)	− (0)	− (28)	− (1.6)	− (0)	− (9.9)	− (0)	− (0.2)	− (12)	− (11.5)	− (5.2)	− (0)	31 (4.2)	9 (0)	4
25	T	− (9.9)	− (7)	− (11.3)	− (15.5)	− (0)	− (11.3)	− (0)	− (8.5)	− (7)	− (8.5)	− (11.3)	− (2.8)	− (2.8)	1 (4.2)	− (0)	0
26	T	− (4.5)	− (0.5)	− (30.2)	− (6.9)	− (0)	− (4)	− (0.5)	− (33.2)	− (13.4)	− (2)	− (1)	− (0)	− (4)	− (0)	− (0)	1
27	T	− (9)	− (3.8)	− (21.2)	− (10.4)	− (0)	− (8.5)	− (2.4)	− (21.7)	− (17.9)	− (3.3)	− (0.5)	− (0)	− (0.9)	− (0)	− (0.5)	2
28	T	− (4.6)	− (0)	− (29)	− (12.2)	− (0)	− (0)	− (0)	− (33.6)	− (17.6)	− (3.1)	− (0)	− (0)	2 (0)	− (0)	− (0)	3
29	T	− (3.8)	− (0.4)	− (5.6)	− (25.2)	− (0)	− (0.4)	− (1.7)	− (2.6)	− (20.1)	3 (24.8)	2 (6.8)	− (0)	6 (3)	9 (5.1)	− (0.4)	1
30	WM	− (16)	− (9.5)	− (0)	− (27.7)	− (2.4)	− (0)	− (8.4)	− (0)	− (0)	− (10.4)	3 (12)	− (5)	− (0)	75 (8.1)	37 (0.6)	16
total cases		0	0	0	0	0	0	0	0	3	27	7	0	8	208	74	88

* see table 2 for breed codes.

† unknown.

Polymorphisms at codons 112 (one of 11 flocks), 141 (four of 11 flocks) and 241 (three of 11 flocks) of the *PrP* gene were identified from cases in flocks, all of which were associated with the ARQ haplotype (table 4). Of particular note, one case was identified in an animal homozygous for phenylalanine at codon 141. All cases in the study flocks were homozygous for proline at codon 168.

**Table 4:** Frequency of polymorphisms at codons 112, 141 and 241 of the *PrP* gene in confirmed scrapie cases.

flock	codon 112				codon 141				codon 241			
	MM	MT	TT	UNK	LL	LF	FF	UNK	PP	PS	SS	UNK
3	7	0	0	7	13	1	0	0	3	0	0	11
16	3	0	0	1	3	1	0	0	3	0	0	1
21	28	1	0	0	12	16	1	0	26	2	0	1
24	25	0	0	3	28	0	0	0	20	7	0	1
30	53	0	0	10	53	10	0	0	54	6	0	3

(Amino acids are: methionine (M); threonine (T); leucine (L); phenylalanine (F); proline (P); serine (S); UNK indicates the number of cases for which information on the polymorphism was not available).

### Age-at-onset

The age-at-onset of disease varied significantly amongst flocks (χ^2^ = 48.1, df = 22, *P* = 0.001) (figure 2d) and amongst genotypes (χ^2^ = 46.4, df = 5, *P*<0.001) (table 5). The earliest mean age-at-onset was in ARQ/ARH animals (although there were only three cases in this genotype, all from the same flock) and VRQ/VRQ animals, followed by ARH/VRQ, ARQ/VRQ, ARQ/ARQ and then ARR/VRQ (table 5). Furthermore, the age-at-onset differed significantly amongst flocks in ARQ/VRQ (*P*<0.001; figure 2e) and ARH/VRQ (*P* = 0.04; though only two flocks had cases in this genotype) animals, but not in VRQ/VRQ (*P* = 0.65; figure 2f), ARQ/ARQ (*P* = 0.95) or ARR/VRQ (*P* = 0.29) sheep.

**Table 5:** Mean, minimum and maximum age-at-onset of clinical signs (in years) by *PrP* genotype.

	*PrP* genotype					
	ARQ/ARH	ARQ/ARQ	ARR/VRQ	ARH/VRQ	ARQ/VRQ	VRQ/VRQ
mean	2.6	4.4	4.9	3.6	3.8	3.0
minimum	1.8	2.3	2.9	2.2	1.9	1.0
maximum	3.0	7.2	6.8	6.8	8.0	6.7
no. cases	3	27	7	8	208	74

Animals of the ARQ/VRQ genotype had an earlier age-at-onset of clinical signs in flocks which had a higher peak incidence (HR = 1.22, *P*<0.001) or a lower outbreak size (HR = 0.88, *P*<0.001); the age-at-onset was later in commercial compared with pedigree flocks (HR = 0.51, *P* = 0.004). Neither mean incidence (*P* = 0.29) nor outbreak duration (*P* = 0.10) explained differences amongst flocks in age-at-onset in ARQ/VRQ animals. The corresponding analysis was not done for the ARH/VRQ genotype, because there were only eight cases in two flocks.

### Control of disease

The most common method of control reported by farmers was genotyping of animals followed by subsequent selective breeding for the animals most resistant to scrapie (n = 11). Other methods of control were the elimination of the offspring of affected animals from the flock (n = 3); the use of genotyping coupled with the elimination of offspring (n = 4); altering lambing practices to control the disease (removed placental tissue, using lambing pens, lambing in a different field; n = 3); breeding replacements (n = 1); using genotyping whilst eliminating the progeny of affected animals and then breeding replacements (n = 2); and genotyping whilst breeding replacements (n = 1). Two farmers reported using no scrapie control methods at all, and three did not answer the question.

### Seasonality

For the majority of flocks, there was no evidence of a seasonal effect in the occurrence of cases. However, for flocks 21 and 30, cases were not distributed uniformly by quarter (with peak numbers of cases in quarters 2 and 1, respectively; for flock 30 there was an additional peak in quarter 3).

## Discussion

In this paper we have described naturally-occurring outbreaks of classical scrapie in 30 UK sheep flocks, the largest number yet described in detail, and have illustrated the marked variability that exists in their epidemiological characteristics, such as outbreak duration, disease incidence and the *PrP* genotype and age-at-onset of cases. Furthermore, while scrapie strain and the stochastic nature of scrapie dynamics may play important roles in driving epidemiological variability between flocks, we have shown that at least some of this variability is accounted for by differences in the flocks' *PrP* genotype profiles. The importance of flock genotype profile is probably underestimated in this paper as we have used a static estimate based on a single blood sampling event at one time-point within each outbreak. A more dynamic estimate, in which the genotype profile changes over time in response to losses from scrapie, may have more explanatory power. However, it is no longer feasible to collect such data within the European Union, because of the control methods utilised against scrapie.

Many modelling studies of scrapie have assumed that outbreak duration would be affected by both flock size and the frequency of *PrP* genotypes within a flock [20], [23], [27], [28]. The results from this study appear at first sight to contradict these assumptions, in that outbreak duration was not associated with flock susceptibility and only marginally associated with flock size. It is likely, however, that the outbreaks were truncated once farmers were given information on the *PrP* genotypes in their flocks, as no cases occurred in animals born after they received the genotype results (figure 1b). Hence, our measure of outbreak duration is likely to be shorter than might have occurred in flocks not under study.

The nature of the study design means that this was unavoidable. Although there were strong scientific grounds for not providing farmers with *PrP* genotypes, there was an obligation to do so for three compelling reasons: (i) Home Office regulations would only allow the sheep to be sampled for surveillance and, therefore, required farmers to be informed of any high risk animals; (ii) it helped to incentivise the farmers, because in the absence of *PrP* genotype information they would have received no benefits from taking part in the study; (iii) it allowed control of when farmers received this information. If genotype information had not been provided, some farmers may have sought *PrP* genotypes from other sources and not informed those running the study. Once farmers received their genotype results, they were able to effectively terminate the epidemic in their flocks by removal of individuals genetically-susceptible to scrapie. As a consequence, no cases occurred in birth cohorts born after farmers were given their genotype results (figure 1b). At the start of the trial, there was a lack of awareness that providing farmers with genotypes would lead to such rapid truncation of their outbreaks; indeed, this is one of the findings of this paper.

There were often gaps of several months between cases being reported within a flock, and in certain examples these gaps exceeded two years (figure 1a). Such long periods without cases being detected suggests that cases may be going undetected or that infection has persisted in the flock without any animals developing clinical disease. An alternative possibility is that such flocks may have experienced more than one scrapie outbreak. We consider this unlikely, given the low flock-level incidence of scrapie in the UK, but note that in one flock (flock 15), a case was confirmed in a purchased animal two years after the previous confirmed case; this animal could have acquired infection in its source flock.

The median incidence (1.1 cases per hundred sheep per year) in the 30 outbreaks presented in this paper is higher than a published estimate for all flocks in the UK which report scrapie (median 0.62; [21]). The range of incidences was similar to that reported in the 1998 and 2002 postal surveys [29], [30] but, as before, higher incidences were more common in this study. By contrast, the median peak incidence (1.8) was much lower than that previously reported for four outbreaks in research flocks (5.0; [17]). These differences probably reflect, on the one hand, a slight tendency for owners of higher-than-average incidence flocks to have approached IAH for inclusion in our study; but on the other hand, for such flocks to have less scrapie than flocks maintained specifically for the study of the disease. Accordingly, while the large number of cases in research flocks facilitates the study of scrapie dynamics in great detail, such outbreaks are in some sense exceptional and, hence, it may be difficult to extrapolate from these outbreaks to all flocks which report scrapie.

Although cases occurred in six *PrP* genotypes (out of the 15 definable in the UK at codons 136, 154 and 171), more than four out of five cases were confined to just two: ARQ/VRQ and VRQ/VRQ. This pattern is similar to that previously described for reported cases [21] and for outbreaks in individual flocks [11]–[13], [17]. However, there was one flock (flock 21) which had markedly different affected genotypes. This flock had a large number of ARQ/ARQ and ARQ/ARH cases, despite having a similar underlying genotype profile to other flocks of the same breed (table 3). The *PrP* genotype of the cases potentially reflects the impact of scrapie strain [31]. While in general cases may be expected to occur only in a limited and predictable number of genotypes dependent upon the underlying flock genotype profile, there are exceptional flocks where this is not the case. A second flock, flock 29, also had a rather different pattern of affected genotypes (although less markedly than flock 21), most notably the occurrence of several ARH/VRQ cases, but also cases in the ARQ/ARQ and ARR/VRQ genotypes (figure 2b,c; table 3). These patterns reflect, first, the high frequency of the ARH allele in the Texel breed such that scrapie occurs in the highly susceptible ARH/VRQ genotype and, second, a tendency for scrapie to affect a broad range of genotypes in Texel sheep [9], [32].

The recent identification of associations between a polymorphism at codon 141 and the risk of atypical scrapie [33]–[35] and between codons 137 [36] and 168 [37] and the risk of BSE in sheep, has raised interest in the polymorphisms found in cases at codons other than 136, 154 and 171. Although the frequencies were too small to identify any differences in risk, we identified cases with polymorphisms at codons 112, 141 and 241, but not at codons 137 or 168 (table 4). Of particular note, there was one case that was homozygous for phenylalanine at codon 141, the genotype associated with a very high risk of atypical scrapie [38], and 28 that were heterozygous at this codon. Accordingly, while F_141_ may be associated with susceptibility to atypical scrapie, it is not associated with resistance to classical scrapie. While we propose no direct link, it is possibly of interest to note that the single flock that was clearly affected by an ARQ-type scrapie (flock 21) was the same flock which showed the greatest level of diversity at *PrP* codons other than 136, 154 and 171 (table 4).

The age-at-onset of clinical disease differed significantly amongst flocks, with the mean ranging from 2.0 to 5.7 years (figure 2d). Given the significant differences in age-at-onset amongst genotypes (table 5), an obvious cause of flock differences would be differing age and *PrP* genotype profiles. However, further analysis suggested that the age-at-onset, at least in animals of the ARQ/VRQ genotype, may be influenced by other factors: for instance, flock husbandry, where the age-at-onset was earlier in purebred than in commercial flocks, and infectious load, where the age-at-onset was earlier in flocks with a higher peak incidence. Although significant differences in the age-at-onset amongst flocks have been reported previously for four flocks [17], the range for mean age-at-onset was much narrower (2.4–2.9 years), despite very large differences in incidence.

Seasonal variation in the number of cases was detected in two (21 and 30) out of the 30 flocks and, in both, the peak number of cases coincided with lambing time in the flock. For the remaining flocks, no seasonality was detected, but the relatively small numbers of cases in most flocks makes any patterns difficult to discern. Previous studies of scrapie-affected flocks have found evidence for seasonality in the number of cases, but in most instances the seasonal peaks did not correspond with lambing time in the flocks [12], [17], [39]. Consequently, analyses of these outbreaks have suggested that seasonality in cases is driven by seasonality in exposure to infectivity [12], [39], which is likely to be greatest at lambing time [14], [40]. However, this requires a reasonably consistent IP; otherwise, the effects of seasonal transmission would be lost because of the effects of a variable IP distribution. The variability in the age-at-onset for our study flocks (figure 2d) suggests that any seasonality in transmission will be lost and, hence, the seasonality in cases, and, in particular, the coincidence of peak cases and lambing, may reflect other mechanisms. For example, stress associated with lambing could lead to the onset of clinical disease; or flocks are likely to be under closer observation at lambing time and, hence, a farmer is more likely to spot clinical signs.

The purchase of infected animals has often been cited as the principal mechanism by which a flock acquires scrapie [41]–[44]. The current study provides supporting evidence for the role played by buying-in infected sheep in the acquisition of scrapie, as purchased animals formed a higher proportion of first compared with subsequent cases. Nevertheless, two-thirds of farmers believed that the first cases in their flocks were in homebred, not purchased animals. This may reflect the difficulty in diagnosing scrapie, such that the first identified case is not necessarily the true first case in the flock. Indeed, there is evidence that farmers become more adept at spotting scrapie in their animals during an outbreak [11]. Alternatively, it may be a consequence of the existence of other yet to be described mechanisms for the introduction of scrapie into a flock.

This study presents a large-scale study of scrapie in its natural setting, involving *PrP* genotype data for nearly 8600 animals and over 400 scrapie cases spread across 30 scrapie-affected farms. We have identified flock-level variation in the age of cases of certain *PrP* genotypes (ARQ/VRQ and ARH/VRQ); we found that the age-at-onset of clinical signs in ARQ/VRQ animals was likely to be earlier in flocks with a higher peak incidence and lower outbreak size of scrapie, and likely to be later in commercial than purebred flocks; we found evidence for the buying-in of scrapie-affected animals being an important means of introducing disease to a flock; we found some evidence that flock-level *PrP* genotype profile and flock size account for variation in some measures of scrapie outbreak size; we identified seasonality associated with lambing time in certain flocks; and we found that a certain flock which was affected by an ARQ-type scrapie also had the greatest level of diversity at *PrP* codons other than 136, 154 and 171.

## References

[1] Parry HB, Oppenheimer DR (1983). Scrapie disease in sheep: historical, clinical, epidemiological, pathological and practical aspects of the natural disease;.

[2] Woolhouse MEJ, Coen P, Matthews L, Foster JD, Elsen JM (2001). A Centuries-long epidemic of scrapie in British sheep?. Trends Microbiol.

[3] Houston EF, Gravenor MB (2003). Clinical signs in sheep experimentally infected with scrapie and BSE.. Vet Rec.

[4] Benestad SL, Sarradin P, Thu J, Schonheit J, Tranulis MA (2003). Cases of scrapie with unusual features in Norway and designation of a new type, Nor98.. Vet Rec.

[5] Dawson M, Hoinville LJ, Hosie BD, Hunter N (1998). Guidance on the use of *PrP* genotyping as an aid to the control of clinical scrapie.. Vet Rec.

[6] Baylis M, Chihota CM, Stevenson E, Goldmann W, Smith A (2004). Risk of scrapie in British sheep of different prion protein genotype.. J Gen Virol.

[7] Goldmann W, Hunter N, Smith G, Foster J, Hope J (1994). *PrP* genotype and agent effects in scrapie: change in allelic interaction with different isolates of agent in sheep, a natural host of scrapie.. J Gen Virol.

[8] Tongue SC, Pfeiffer DU, Warner R, Elliot H, del Rio Vilas V (2006). Estimation of the relative risk for developing clinical scrapie: the role of prion protein (*PrP*) genotype and selection bias.. Vet Rec.

[9] Baylis M, Goldmann W, Houston F, Cairns D, Chong A (2002). Scrapie epidemic in a fully *PrP*-genotyped sheep flock.. J Gen Virol.

[10] Baylis M, Houston F, Goldmann W, Hunter N, McLean AR (2000). The signature of scrapie: differences in the *PrP* genotype profile of scrapie-affected and scrapie-free UK sheep flocks.. P Roy Soc B-Biol Sci.

[11] McIntyre KM, Gubbins S, Goldmann W, Stevenson E, Baylis M (2006). The time-course of a scrapie outbreak.. BMC Vet Res.

[12] Diaz C, Vitezica ZG, Rupp R, Andreletti O, Elsen JM (2005). Polygenic variation and transmission factors involved in the resistance/susceptibility to scrapie in a Romanov flock.. J Gen Virol.

[13] Elsen JM, Amigues Y, Schelcher F, Ducrocq V, Andreoletti O (1999). Genetic susceptibility and transmission factors in scrapie: detailed analysis of an epidemic in a closed flock of Romanov.. Arch Virol.

[14] Foster JD, Dickinson AG (1989). Age at death from natural scrapie in a flock of Suffolk sheep.. Vet Rec.

[15] Hunter N, Foster JD, Goldmann W, Stear MJ, Hope J (1996). Natural scrapie in a closed flock of Cheviot sheep occurs only in specific *PrP* genotypes.. Arch Virol.

[16] Hunter N, Moore L, Hosie BD, Dingwall WS, Greig A (1997). Association between natural scrapie and *PrP* genotype in a flock of Suffolk sheep in Scotland.. Vet Rec.

[17] Redman CA, Coen PG, Matthews L, Lewis RM, Dingwall WS (2002). Comparative epidemiology of scrapie outbreaks in individual sheep flocks.. Epidemiol Infect.

[18] Anderson RM, May RM (1991). Infectious diseases of humans. Dynamics and control.

[19] Hagenaars TJ, Donnelly CA, Ferguson NM, Anderson RM (2003). Dynamics of a scrapie outbreak in a flock of Romanov sheep - estimation of transmission parameters.. Epidemiol Infect.

[20] Hagenaars TJ, Ferguson NM, Donnelly CA, Anderson RM (2001). Persistence patterns of scrapie in a sheep flock.. Epidemiol Infect.

[21] del Rio Vilas VJ, Guitian J, Pfeiffer DU, Wilesmith JW (2006). Analysis of data from the passive surveillance of scrapie in Great Britain between 1993 and 2002.. Vet Rec.

[22] Goldmann W, Baylis M, Chihota C, Stevenson E, Hunter N (2005). Frequencies of *PrP* gene haplotypes in British sheep flocks and the implications for breeding programmes.. J Appl Microbiol.

[23] Gubbins S (2005). A modelling framework to describe the spread of scrapie between sheep flocks in Great Britain.. Prev Vet Med.

[24] Gubbins S, Roden JA (2006). Breeding programmes for TSE resistance in British sheep - II. Assessing the impact on the prevalence and incidence of scrapie.. Prev Vet Med.

[25] Collett D, Chatfield C, Zidek JV (1994). Modelling survival data in medical research;.

[26] Sokal RR, Rohlf FJ (1981). Biometry: principles and practice of statistics in biological research.

[27] Matthews L, Woolhouse MEJ, Hunter N (1999). The basic reproduction number for scrapie.. P Roy Soc B-Biol Sci.

[28] Woolhouse MEJ, Stringer SM, Matthews L, Hunter N, Anderson RM (1998). Epidemiology and control of scrapie within a sheep flock.. P Roy Soc B-Biol Sci.

[29] Hoinville LJ, Hoek A, Gravenor MB, McLean AR (2000). Descriptive epidemiology of scrapie in Great Britain: results of a postal survey.. Vet Rec.

[30] Sivam K, Baylis M, Gravenor MB, Gubbins S (2006). Descriptive analysis of the results of an anonymous postal survey of the occurrence of scrapie in Great Britain in 2002.. Vet Rec.

[31] Baylis M, Goldmann W (2004). The genetics of scrapie in sheep and goats.. Curr Mol Med.

[32] Belt PBGM, Muileman IH, Schreuder BEC, Ruijter JB-d, Gielkens ALJ (1995). Identification of five allelic variants of the sheep *PrP* gene and their association with natural scrapie.. J Gen Virol.

[33] Lühken G, Buschmann A, Groschup MH, Erhardt G (2004). Prion protein allele A(136)H(154)Q(171) is associated with high susceptibility to scrapie in purebred and crossbred German Merinoland sheep.. Arch Virol.

[34] Moum T, Olsaker I, Hopp P, Moldal T, Valheim M (2005). Polymorphisms at codons 141 and 154 in the ovine prion protein gene are associated with scrapie Nor98 cases.. J Gen Virol.

[35] Saunders GC, Cawthraw S, Mountjoy SJ, Hope J, Windl O (2006). *PrP* genotypes of atypical scrapie cases in Great Britain.. J Gen Virol.

[36] Vaccari G, D'Agostino C, Nonno R, Rosone F, Conte M (2007). Prion protein alleles showing protective effect on the susceptibility of sheep to scrapie and BSE.. J Virol.

[37] Goldmann W, Houston F, Stewart P, Perucchini M, Foster J (2006). Ovine prion protein variant A(136) R(154)L(168)Q(171) increases resistance to experimental challenge with bovine spongiform encephalopathy agent.. J Gen Virol.

[38] European Food Safety Authority (2005). Opinion of the scientific panel on biological hazards on the request from the European Commission on classification of atypical transmissible spongiform encephalopathy (TSE) cases in small ruminants.. The EFSA Journal.

[39] Touzeau S, Chase-Topping ME, Matthews L, Lajous D, Eychenne F (2006). Modelling the spread of scrapie in a sheep flock: evidence for increased transmission during lambing seasons.. Arch Virol.

[40] Hunter N, Cairns D (1998). Scrapie-free Merino and Poll Dorset sheep from Australia and New Zealand have normal frequencies of scrapie-susceptible *PrP* genotypes.. J Gen Virol.

[41] Healy AM, Morgan KL, Hannon D, Collins JD, Weavers E (2004). Postal questionnaire survey of scrapie in sheep flocks in Ireland.. Vet Rec.

[42] Hopp P, Ulvund MJ, Jarp J (2001). A case-control study on scrapie in Norwegian sheep flocks.. Prev Vet Med.

[43] McIntyre KM, Gubbins S, Sivam KS, Baylis M (2006). Flock-level risk factors for scrapie in Great Britain: analysis of a 2002 anonymous postal survey.. BMC Vet Res.

[44] McLean AR, Hoek A, Hoinville LJ, Gravenor MB (1999). Scrapie transmission in Britain: a recipe for a mathematical model.. P Roy Soc B-Biol Sci.

