# Correction: Epidemiological Characteristics of Classical Scrapie Outbreaks in 30 Sheep Flocks in the United Kingdom

**DOI:** 10.1371/annotation/adaf5807-7a9a-4132-9dd9-ccc0b1c2110a

**Published:** 2009-01-12

**Authors:** K. Marie McIntyre, Simon Gubbins, Wilfred Goldmann, Nora Hunter, Matthew Baylis

The second affiliation appears incorrectly. It should read: Neuropathogenesis Division, The Roslin Institute and R(D)SVS, University of Edinburgh, Edinburgh, United Kingdom

